# Transient intussusception following single anastomosis duodeno-ileal (SADI) bypass surgery: case report and literature review

**DOI:** 10.1093/jscr/rjag860

**Published:** 2026-09-29

**Authors:** Kaeun Bae, Fraser Hugh Simpson, Michael Hatzifotis

**Affiliations:** Queensland Health, General Surgery Department, 199 Ipswich Rd, Woolloongabba QLD 4102, Australia; Queensland Health, General Surgery Department, 199 Ipswich Rd, Woolloongabba QLD 4102, Australia; Queensland Health, General Surgery Department, 199 Ipswich Rd, Woolloongabba QLD 4102, Australia

**Keywords:** intussusception, SADI bypass, bariatric surgery, Roux-en-Y gastric bypass, small bowel obstruction

## Abstract

Intussusception is a rare but recognized complication following bariatric surgery, most commonly occurring after a Roux-en-Y gastric bypass. This report details a case of postoperative transient intussusception following a single anastomosis duodeno-ileal (SADI) bypass which has not previously been described. A 47-year-old male presented with intermittent colicky abdominal pain with a computed tomography demonstrating features consistent with intussusception several years after a SADI bypass. The patient underwent diagnostic laparoscopy which showed the intussusception had spontaneously resolved. Intussusception following bariatric surgery is uncommon, with proposed mechanisms, including altered bowel motility or loss of coordinated bowel peristalsis, loss of mesenteric fat after significant weight loss, and anastomotic staple lines acting as potential points of intussusception. Although exceedingly rare, intussusception should be considered in the differential diagnosis of post-SADI bypass patients presenting with episodic abdominal pain.

## Introduction

Bariatric surgery has been commonly utilized for its efficient management of morbid obesity and its associated comorbidities. The single anastomosis duodeno-ileal (SADI) bypass, a modification of the duodenal switch, involves a single duodeno-ileal anastomosis without the second jejunal anastomosis that the Roux-en-Y gastric bypass (RYGB) contains. Intussusception, defined as the telescoping of one segment of the intestine into another, is an uncommon complication post-bariatric surgery but can present with significant morbidity if unrecognized.

Intussusception is an uncommon cause of small bowel obstruction in adults, accounting for approximately 1%–5% of cases [1]. It may be physiological, asymptomatic and diagnosed incidentally with cross sectional imaging or at laparoscopy performed for other reasons [1–6]. It may also be symptomatic and diagnosed radiologically with cross sectional imaging or during laparoscopy.

Transient intussusception has been reported following RYGB, with a concerning target sign on cross-sectional imaging but no evidence of intussusception on laparoscopy [6]. Transient intussusception has not been reported after the SADI bypass. This report highlights a case of transient intussusception following SADI bypass and reviews the literature concerning intussusception after SADI.

## Case report

A 47-year-old male presented to a hospital emergency department with two days of colicky abdominal pain and bowel changes. He denied any melaena or haematochezia and reported normal bowel habits prior to this event. On examination, his abdomen was mildly distended and generally tender without peritonism. He had a sleeve gastrectomy 10 years previously, which was subsequently revised with a SADI bypass. He also had a previous appendicectomy and prostatectomy. He was a non-smoker, denied alcohol use, and was otherwise fit and healthy with no regular medications. He had no significant dietary changes.

He underwent a contrast-enhanced computed tomography (CT) of the abdomen and pelvis which demonstrated a classic ‘target sign’ consistent with small bowel intussusception involving the common channel distal to the anastomosis, with no evidence of bowel ischemia or perforation (Figs 1–3).

**Figure 1:**
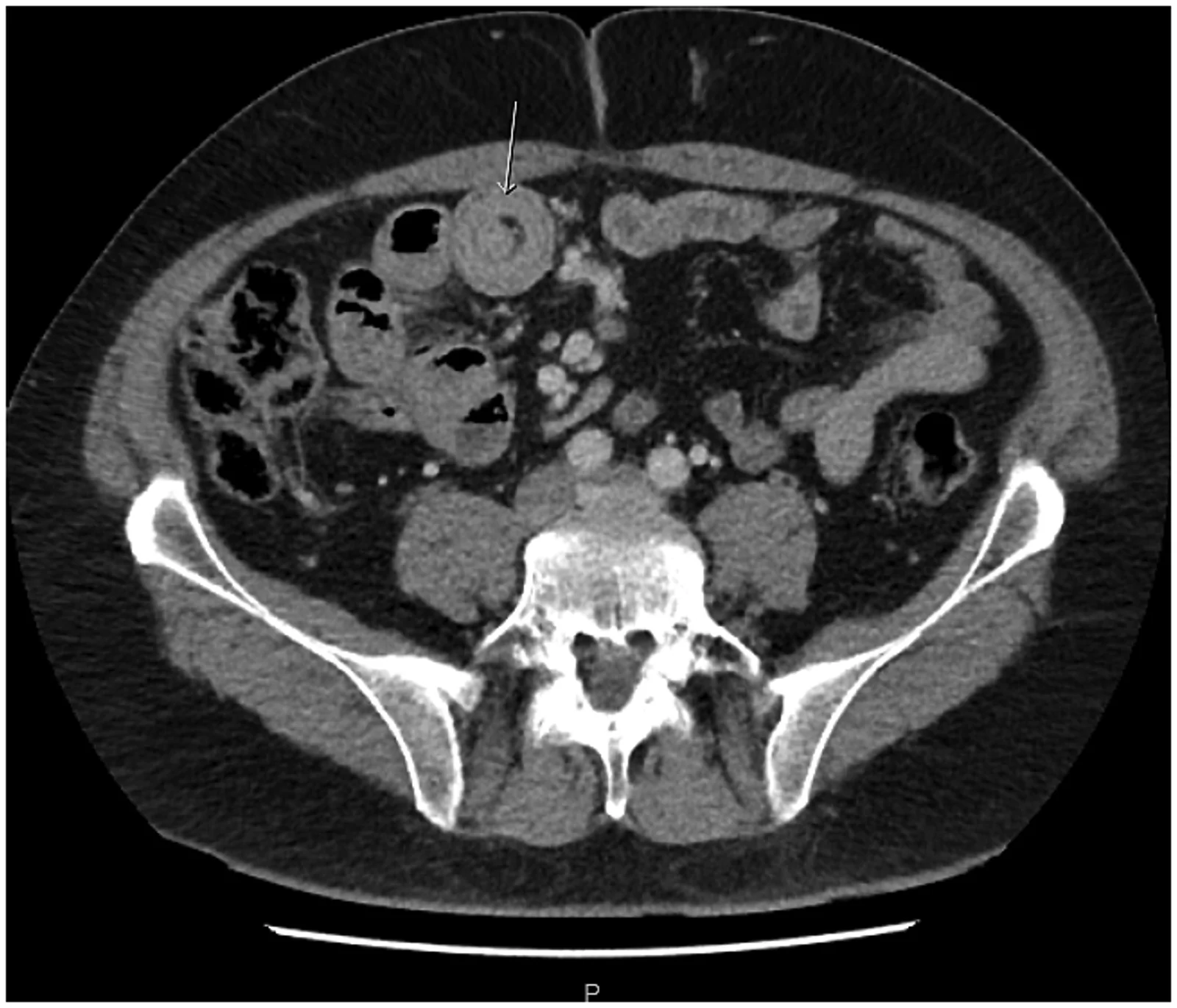
Contrast CT axial view showing ‘target’ sign, indicated by the arrow.

**Figure 2:**
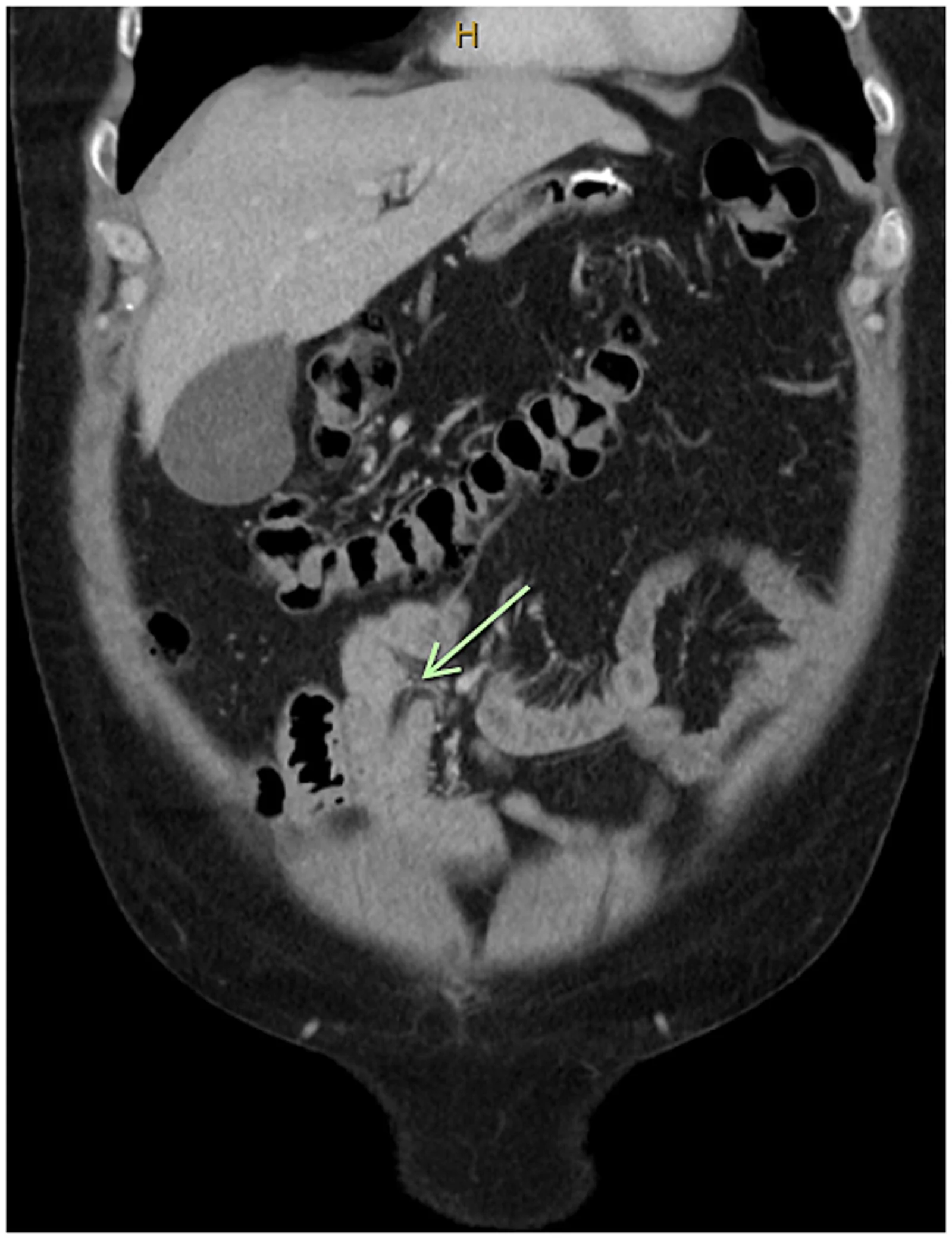
Contrast CT coronal view showing the site of intussusception, indicated by the arrow.

**Figure 3:**
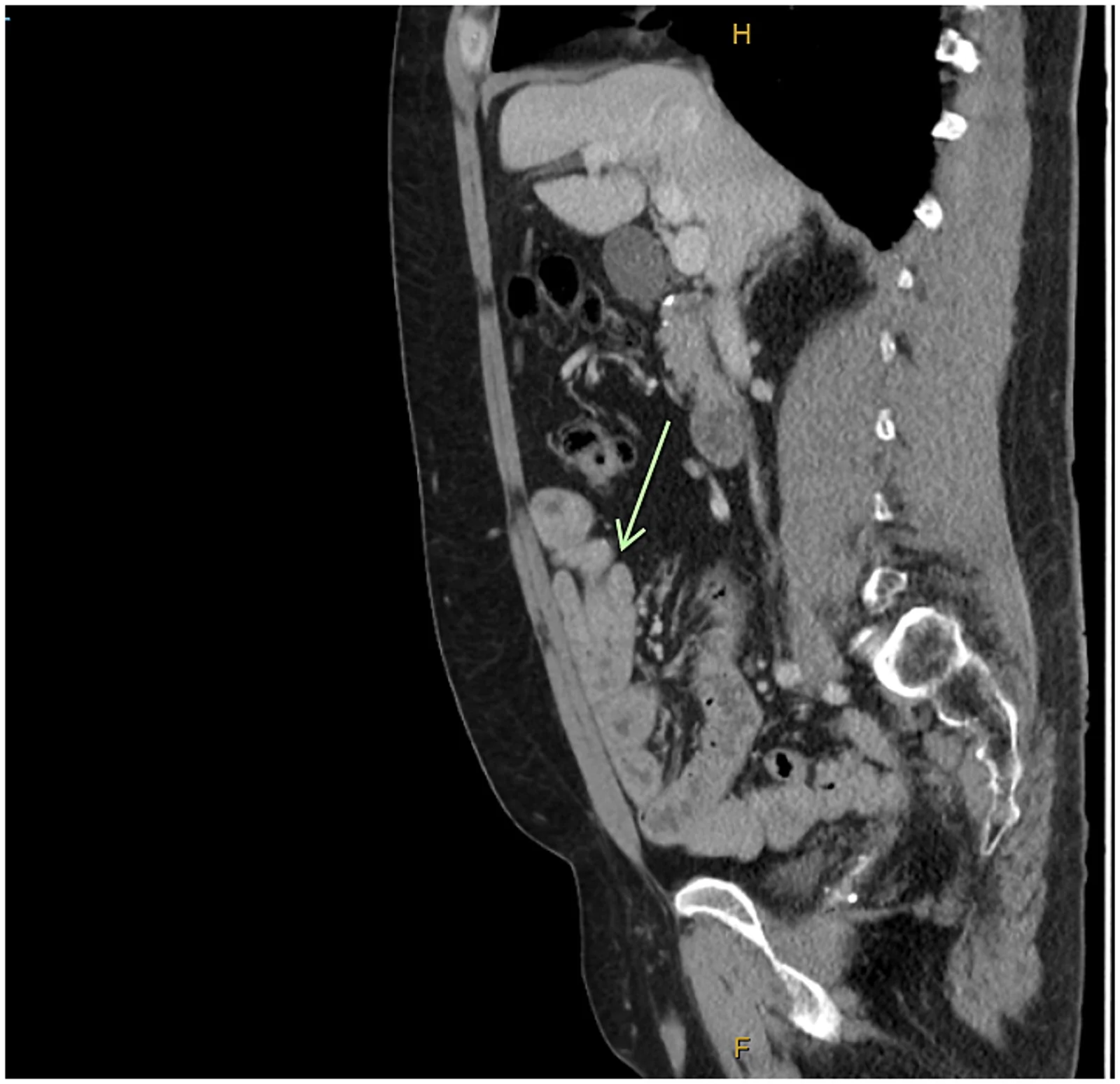
Contrast CT sagittal view showing the site of intussusception, indicated by the arrow.

Laboratory investigations revealed normal white cell count and mildly elevated C-reactive protein (CRP) (Table 1).

**Table 1:** Laboratory investigations.

Marker	Patient level	Reference level	Reference unit
CRP	37	<5	mg/L
Haemoglobin	141	135–175	g/L
White cell count	5.4	3.5–10.0	10^9^/L
Potassium	4.7	3.5–5.5	Mmol/L
INR	1.2	0.8–1.2	
APTT	27	23–37	s

The patient was taken for a diagnostic laparoscopy; however, no abnormal bowel was identified, and the intussusception had spontaneously resolved. The postoperative course was uneventful, and the patient was discharged on day 1 post-surgery. No further investigations, such as a capsule endoscopy or magnetic resonance enterography, were undertaken to investigate the intussusception as it had spontaneously resolved.

## Discussion

The SADI bypass is a variant of the biliopancreatic diversion with duodenal switch (BPD-DS), involving a sleeve gastrectomy and a single duodeno-ileal anastomosis. The SADI bypass differs anatomically from RYGB in that it does not involve a Roux limb or jejuno-jejunal anastomosis. A literature search using the keywords of ‘intussusception’, ‘SADI bypass’, and ‘bariatric surgery’ was completed on Pubmed which did not yield any reports.

Intussusception post-bariatric surgery is believed to be related to altered bowel motility, changes in mesenteric anatomy, and the creation of new entero-enteric anastomoses [7]. Clinical presentation is often nonspecific, including intermittent abdominal pain, nausea, vomiting, and, less commonly, signs of bowel obstruction. Unlike patients who have intussusception without bariatric surgery, patients post bariatric surgery usually have no pathological lesion in the lumen or wall of the intestine causing the intussusception. It typically occurs in the small intestine either proximal or at the entero-enteric anastomosis; it is uncommon for it to occur in the common channel. It is unclear if this episode of intussusception was purely incidental and not related to altered anatomy post bariatric surgery.

In RYGB patients, management with bowel resection has been associated with lower recurrence rates compared to reduction alone [4]. Intussusception can occur as early as at the time of the RYGB procedure or any time after the procedure. The reported incidence after RYGB ranges from 0.07% to 0.64%, based on retrospective series and pooled analyses [2, 4].

To our knowledge, transient intussusception following SADI bypass, as seen in this patient, has not previously been reported. The underlying mechanism may be like that proposed after RYGB, although the risk is likely lower because SADI bypass does not involve formation of a jejuno-jejunal anastomosis. Electrolyte disturbances and weight loss may also contribute to altered bowel motility.

When diagnosed on cross sectional imaging, often patients will proceed to diagnostic laparoscopy. At laparoscopy, the intussusception may have spontaneously resolved in which case no further intervention is usually required [8]. The dilemma for many surgeons is whether resection is required after reduction of the intussusception where the small intestine appears healthy and viable. Pexy of the involved limb of the intestine to prevent further intussusception has been described, however the effectiveness of this is undetermined [3].

The decision on how to proceed after diagnosing intussusception at laparoscopy remains with the treating surgeons and depends on several factors such as the patient’s clinical scenario, appearance at laparoscopy, viability of the small intestine, position of the intussusception in relation to the entero-enteric anastomosis, and the likelihood of recurrence. If a patient fails to clinically improve after a trial of conservative management, it may be more likely that the intussusception has not resolved and thus puts bowel at risk of ischaemia or necrosis. It is unclear if certain configurations of the terminal-lateral anastomosis have a higher likelihood of leading to intussusception or whether this anastomosis should be revised in patients undergoing resection of small intestine for intussusception post RYGB.

This case represents a rare presentation of intussusception following SADI. Although not previously well documented, intussusception should be considered in SADI patients presenting with intermittent abdominal pain or signs of small bowel obstruction. Awareness of this potential complication may facilitate earlier diagnosis and management. Large outcomes and systematic reviews of SADI have not reported intussusception as a recognized complication, suggesting an extremely low incidence or underreporting. As SADI becomes more widely adopted, rare complications may become increasingly apparent. Clinicians need to be aware of transient intussusception as conservative management may be possible in selected cases.
